# Tiotropium and Fluticasone Inhibit Rhinovirus-Induced Mucin Production via Multiple Mechanisms in Differentiated Airway Epithelial Cells

**DOI:** 10.3389/fcimb.2020.00278

**Published:** 2020-06-19

**Authors:** Ying Wang, Dennis K. Ninaber, Annemarie van Schadewijk, Pieter S. Hiemstra

**Affiliations:** Department of Pulmonology, Leiden University Medical Center, Leiden, Netherlands

**Keywords:** rhinovirus, exacerbation, tiotropium, fluticasone, mucin production, airway epithelial cells, SPDEF, purinergic signaling

## Abstract

Human rhinoviruses (HRVs) are associated with acute exacerbations in patients with chronic obstructive pulmonary disease (COPD) and asthma, which are accompanied by mucus hypersecretion. Whereas, various studies have shown that HRVs increase epithelial mucin production and thus may directly contribute to mucus hypersecretion. The effects of drugs used in the treatment of COPD and asthma on HRV-induced mucin production in epithelial cell cultures have not been studied. In the present study, we assessed effects of HRVs on mucin production and secretion in well-differentiated primary human bronchial epithelial cells (PBEC) and studied the effect of the inhaled corticosteroid fluticasone propionate and the long-acting muscarinic antagonist tiotropium bromide on this process. Differentiated PBEC that were cultured at the air-liquid interface (ALI-PBEC) were infected with HRV-A16 and HRV-1B. Quantitative PCR, immunofluorescence staining, ELISA, periodic acid-Schiff (PAS) staining and immunostaining assays were used to assess the effects of HRV infection. Here we demonstrate that both HRV-A16 and HRV-1B increased mucin (MUC5AC and MUC5B) gene expression and protein release. When exploring this in more detail in HRV-A16-infected epithelial cells, mucin expression was found to be accompanied by increases in expression of SAM-pointed domain-containing Ets-like factor (SPDEF) and SPDEF-regulated genes known to be involved in the regulation of mucin production. We also found that pre-treatment with the purinergic P2R antagonist suramin inhibits HRV-enhanced MUC5AC expression and protein release, implicating involvement of purinergic signaling by extracellular ATP. We furthermore found that both fluticasone and tiotropium decreased HRV-induced mucin production without affecting viral replication, and obtained evidence to suggest that the inhibitory effect of fluticasone involved modulation of SPDEF-regulated genes and extracellular ATP release. These data show that both tiotropium and fluticasone inhibit HRV-induced epithelial mucin production independent of viral clearance, and thus provide insight into the mechanisms underlying beneficial effects of tiotropium and fluticasone in the treatment of COPD, asthma and accompanying exacerbations in these patients. Furthermore, our findings provide additional insight into the mechanisms by which HRV increases epithelial mucin production.

## Introduction

The epithelium lining the respiratory tract from the nasal cavity to the terminal bronchioles serves as an important barrier and regulator of host defense and immune responses (Whitsett, 2018). Ciliated, goblet, club and basal cells are the main cell types that constitute the (large) airway epithelium (Hiemstra et al., 2015; Iwasaki et al., 2017). Goblet cells of the surface epithelium, together with the submucosal glands, produce the major gel-forming mucins MUC5AC and MUC5B, which play a central role in normal host defense against infection via mucociliary clearance and antimicrobial properties of mucus (Fahy and Dickey, 2010). In contrast, chronic mucus hypersecretion may impair mucociliary clearance and cause obstruction of especially small airways by mucus plugging, and thus contribute to progression of chronic inflammatory airway diseases such as asthma, chronic obstructive pulmonary disease (COPD) and cystic fibrosis (CF) (Bonser et al., 2016; Ridley and Thornton, 2018; Saco et al., 2018). Therefore, full insight in the regulation of mucin production and goblet cell formation is essential to improve treatment.

A variety of triggers contribute to mucin production, including allergic inflammation, environmental pollutants, viral exposure and bacterial products, and various mechanisms have been implicated in the regulation of mucin production, including signaling via the epidermal growth factor receptor, the Notch pathway and purinergic signaling following extracellular release of adenosine 5'-triphosphate (ATP) (Boucherat et al., 2013; Krishn et al., 2018). At the transcriptional level, SAM-pointed domain-containing Ets-like factor (SPDEF) plays a critical role in regulating goblet cell differentiation and mucin production by interacting with forkhead box protein 3 (FOXA3, also termed HNF3γ), inducing the expression of anterior gradient 2 (AGR2) and suppressing the expression of forkhead box protein A2 (FOXA2) (Chen et al., 2009; Whitsett et al., 2019). Purinergic signaling via extracellular ATP, mediated by P2 purinergic receptors (P2R), was found to contribute to the regulation of mucin production and secretion in airway epithelial cells (Pelleg et al., 2016; Shishikura et al., 2016). *In vitro* culture studies, studies in mouse models and clinical studies have shown that human rhinoviruses (HRVs), frequently associated with COPD and asthma exacerbations, contribute to excessive mucus levels (Hewson et al., 2010; Mallia et al., 2011; Han et al., 2017), which involves SPDEF-regulated genes and extracellular ATP release (Chen et al., 2014; Shishikura et al., 2016). HRVs are positive-sense, single-stranded-RNA, classified as three species (HRV-A, B and C) based no phylogeny (Jacobs et al., 2013). HRV-B and most HRV-A variants (the major group, such as HRV-A16) bind to intercellular adhesion molecule 1 (ICAM-1), a subset of HRV-A types (the minor group, like HRV-1B) use low-density lipoprotein receptor (LDLR) and cadherin-related family member 3 (CDHR3) may be the receptor of HRV-C types (Blaas and Fuchs, 2016).

A variety of treatment strategies used in patients with chronic inflammatory lung diseases have been shown to affect epithelial mucin production. Macrolides such as azithromycin have been reported to prevent exacerbations in COPD patients (Albert et al., 2011), and have been reported to inhibit mucin production in epithelial cells (Mertens et al., 2016). Anticholinergic agents such as the long-acting muscarinic antagonist (LAMA) tiotropium bromide are used in the treatment of COPD and (poorly-controlled) asthma, and have been shown to inhibit mucin production in animal models and in human epithelial culture models using IL-13 and neutrophil elastase as triggers for mucin production (Kistemaker et al., 2015; Komiya et al., 2017). Inhaled corticosteroids (ICS) are also widely used in the treatment of asthma and COPD, but the effects on mucin production observed differ, probably explained by the use of different culture models (cell lines vs. primary cells, patients cells vs. healthy donors) and animal models (Kanoh et al., 2011; Lachowicz-Scroggins et al., 2017; Singanayagam et al., 2018). So far it is unclear to which extent LAMA and ICS control HRV-induced mucin production.

Here, using a model of differentiated primary human airway epithelial cells, we show that the ICS fluticasone propionate inhibits HRV-increased mucin gene expression and protein release, and demonstrate that this is accompanied by modulation of expression of SPDEF-regulated genes and extracellular ATP-mediated purinergic signaling. Tiotropium was found to decrease HRV-induced mucin secretion and goblet cell numbers. These data further increase our understanding of role of inhaled corticosteroids and anticholinergics in the treatment of asthma and COPD exacerbations.

## Methods

### Cell Culture

Primary human bronchial epithelial cells (PBEC) were isolated from tumor-free resected bronchial tissue obtained from lung cancer patients undergoing lobectomy at the Leiden University Medical Center (Leiden, the Netherlands). Use of such lung tissue that became available for research within the framework of patient care was in line with the “Human Tissue and Medical Research: Code of conduct for responsible use” (2011) (www.federa.org), that describes the no-objection system for coded anonymous further use of such tissue. PBEC were cultured at the air-liquid interface (ALI) for 21 days to achieve mucociliary differentiation as previously described (Schrumpf et al., 2020). In brief, 40,000 cells at passage 2 were seeded on 12-well transwell membranes (Corning Costar, Cambridge, MA, USA) which were coated with 10 μg/ml human fibronectin (Sanbio, Uden, the Netherlands), 30 μg/ml PureCol (Advanced BioMatrix, CA, USA) and 10 μg/ml bovine serum albumin (Fraction V; Thermo Fisher Scientific, Carlsbad, CA, USA) in a 1:1 mixture of Bronchial Epithelial Cell Medium-basal (BEpiCM-b; ScienCell, Sanbio) and Dulbecco's modified Eagle's medium (DMEM) (Stemcell Technologies, Köln, Germany). This medium contains 12.5 mM Hepes, bronchial epithelial cell growth supplement, 100 U/ml penicillin, 100 ug/ml streptomycin (all from ScienCell), 2 mM glutaMAX (Thermo Fisher Scientific) and 1 nM of the retinoic acid receptor agonist EC23 (Tocris, Abingdon, UK). After confluence was reached, the apical medium was removed and cells were cultured at the air-liquid interface in complete medium with 50 nM EC23 for 21 days and medium was refreshed and apical side was washed three times a week. High trans-epithelial electrical resistance (TEER>500 Ω·cm^2^), visible cilia beating and mucus release appeared during mucociliairy differentiation. In selected experiments, cell differentiation was skewed toward increased production of ciliary cells by inhibition of Notch signaling using DAPT as described in the Supplementary Material.

### Viral Stocks

Human rhinovirus type 16 (HRV-A16, major group, ATCC VR-283™, Wesel, Germany) and 1B (HRV-1B, minor group, ATCC VR-1645™) viral stocks were prepared in H1-HeLa cells (ATCC CRL-1958™). Cells were proliferated in DMEM medium containing 10% v/v Fetal Bovine Serum (FBS, Thermo Fisher Scientific), 2% v/v 1 M Hepes and 0.075% w/v NaHCO_3_ (Thermo Fisher Scientific). Viral stocks from ATCC infected cells in the presence of 2% FBS for about 24 h and when a cytopathic effect appeared. After removing the supernatant, D-PBS (Thermo Fisher Scientific) was added cells were lysed via freezing/thawing twice. After that, viruses were obtained by centrifuging and filtering. Viral stocks from pre-cultures were further used to amplify numbers through a second culture. Similarly, the supernatant with virus is obtained. To make a total of 40 ml 4 ml of 5 M NaCl (Boom, Meppel, the Netherlands)was added, 2.8 g polyethyleneglycol (PEG) 6000 (VWR, Amsterdam, the Netherlands) was added to precipitate the virus; pellets were resuspended in PBS, filtered and concentrated using 0.2 micron filter Amicon spin column (Millipore, Bedford, MA, USA). HRV stocks were aliquoted and stored at −80°C until use. Infectivity was determined by 50% tissue culture infective dose (TCID50) measurements. The multiplicity of infection (MOI) was varied according to the requirements of the experiment.

To generate replication-deficient HRVs, stocks of purified HRV-16 were exposed for about 20 min to an ultra-violet (UV) transilluminator (Stratagene, La Jolla, California) at specific wavelength. HRV-deficient replication was confirmed by TCID50 measurements and PCR analysis. UV-treated viruses were used in experiments at doses identical to those for intact (replication competent) HRVs in all experiments.

### Rhinovirus Infection

Differentiated ALI-PBEC were incubated for 24 h in hydrocortisone (HC)-free complete medium before infection with HRV by addition of virus in 100 μL PBS with gentle room temperature shaking. After 1 h, the apical fluid was removed and cells were washed three times with PBS, and fresh HC-free medium was added to the basal compartment. Cultures were harvested at 24, 48, or 72 h after infection by collection of apical wash, basal medium and lysis of cells for isolation of RNA. Apical washes were obtained by adding 200 μL PBS to the apical side for 10 min. In selected experiments, ALI-PBEC were infected by HRV-A16 or HRV-1B at different MOI (0.1, 1, 5). Cytotoxicity was assessed using an LDH assay (Supplementary Material).

### Experimental Treatments

In experiments evaluating the effect of tiotropium (a gift from Boehringer Ingelheim, Ingelheim Germany) or fluticasone (Sigma-Aldrich, Zwijndrecht, The Netherlands) on HRV-induced mucin production, cells were pre-treated by incubation in HC-free complete medium containing tiotropium (10–1,000 nM) or fluticasone (10–1,000 nM) prior to infection with HRV. After infection, the basal medium was replaced by fresh free-HC medium containing tiotropium or fluticasone.

In experiments aimed to investigate the role of extracellular ATP release in virus-induced mucin production, suramin (a non-selective P2R antagonist, TOCRIS) was added to basal media at 10 μM for 30 min prior to HRV treatment. To investigate the ability of tiotropium and fluticasone to inhibit ATP- induced mucin expression, cells pre-treated with tiotropium/fluticasone were stimulated with 100 μM ATP at the basal side for 24 h.

In experiments with DAPT (γ-secretase inhibitor, TOCRIS), ALI-PBEC were incubated in complete medium supplemented with either 5 μM DAPT or solvent control (0.1% DMSO) during differentiation for 14 days. On day 14, cells were infected with HRV-A16 at MOI 1, and following infection the basal medium was replaced by fresh HC-free medium containing 5 μM DAPT or solvent control and cells were incubated for another 24 h.

### Extraction of Total RNA, mRNA Quantification and Quantitative Real-Time PCR (qPCR)

Cells were lysed using RNA lysis buffer from Promega, Leiden, the Netherlands. Total RNA was robotically isolated using Maxwell® 16 simply RNA tissue kit (Promega) and quantified using a Nanodrop ND-1000 UV-visible spectrophotometer (Nanodrop Technologies, Wilmington, DE, USA). RNA was reverse-transcribed and cDNA was amplified by qPCR (Bio-Rad, Veenendaal, the Netherlands). Relative gene expression compared to reference genes Ribosomal Protein L13a (RPL13A) and ATP synthase, H^+^ transporting, mitochondrial F1 complex, beta polypeptide (ATP5B) were calculated according to the standard curve method. Reference genes were selected out of 8 candidate reference genes using the “Genorm” software (Genorm; Primer Design, Southampton, UK). Primer pairs are presented in Table 1.

**Table 1:** Primer sequences.

**Gene**	**Forward primer**	**Reverse primer**
MUC5AC	5′-CCTTCGACGGACAGAGCTAC-3′	5′-TCTCGGTGACAACACGAAAG-3′
MUC5B	5′-GGGCTTTGACAAGAGAGT-3′	5′-AGGATGGTCGTGTTGATGCG-3′
FOXA3	5′-CACTCGCTGTCTTTCAACGAC-3′	5′-AGACCCTGTCCCGTTCCTG-3′
FOXJ1	5′-GGAGGGGACGTAAATCCCTA-3′	5′-TTGGTCCCAGTAGTTCCAGC-3′
AGR2	5′-GGGGTGACCAACTCATCTGG-3′	5′-AGGAGGACAAACTGCTCTGC-3′
LDLR	5′-CGTGCTCCTCGTCTTCCTTT-3′	5′-TCTGTCTCGAGGGGTAGCTG-3′
ICAM-1	5′-ATGCCCAGACATCTGTGTCC-3′	5′-GGGGTCTCTATGCCCAACAA-3′
HRV-1B	5′-CCATCGCTCACTATTCAGCAC-3′	5′-TCTATCCCGAACACACTGTCC-3′
HRV-A16	5′-ACCCTCAATACATACGCCAACT-3′	5′-TTCCAAGCCATCCATTCCA-3′
SCGB1A1	5′-ACATGAGGGAGGCAGGGGCTC-3′	5′-ACTCAAAGCATGGCAGCGGCA-3′
SPDEF	5′-ATGAAAGAGCGGACTTCACCT-3′	5′-CTGGTCGAGGCACAGTAGTG-3′
ATP5B	5′-TCACCCAGGCTGGTTCAGA-3′	5′-AGTGGCCAGGGTAGGCTGAT-3′
RPL13A	5′-AAGGTGGTGGTCGTACGCTGTG-3′	5′-CGGGAAGGGTTGGTGTTCATCC-3′

### Mucin 5AC/5B Protein Measurements by ELISA

To assess mucin secretion, apical washes of epithelial cells were performed by adding 200 μL PBS to the apical surface followed by 10 min incubation at 37°C and subsequent collection of the apical wash. Levels of Mucin 5AC and Mucin 5B protein in the apical washes were measured by enzyme-linked immunoassays (ELISA) and expressed as arbitrary units/mL (AU/mL) calculated based on a standard line constructed using sputum samples, essentially as previously described (Mertens et al., 2017).

### PAS Staining

Transwells with cells were rinsed using PBS, and 4% (w/v) paraformaldehyde (Sigma-Aldrich) in PBS was added into basal and apical compartments and incubated during 10 min at 4°C. Membranes were washed and stored in PBS at 4°C until use. Membranes were embedded in 2% (w/v) agar (VWR) prior to paraffin embedding and cut into 4 μm thick slices. For periodic acid-Schiff (PAS) staining, sections of agar-paraffin embedded cell cultures were deparaffinised, treated with 0.5% (w/v) periodic acid (Sigma-Aldrich), then stained with Schiff's reagent (Sigma-Aldrich) and counterstained with haematoxyline (VWR) to detect goblet cells.

### Histochemistry

For immunostaining, deparaffinised sections were blocked for endogenous peroxidase using methanol/0.3% (v/v) H_2_O_2_ and rehydrated later. Mucin-positive cells were stained with a Mucin 5AC antibody (1:200; 45M1; Thermo Fisher Scientific) and then incubated with DAKO EnVision-HRP. Vector® NovaRED™ Substrate kit (VECTOR, Burlingame, CA, USA) was used as suitable chromogen. Cells were counted as the number of Mucin 5AC-positive cells per length basal membrane in duplicate in a blinded fashion.

### Immunofluorescence Staining and Confocal Microscopy

Transwell membranes with cells were rinsed using PBS and 4% (w/v) paraformaldehyde in PBS was added into basal and apical compartments and incubated during 30 min at room temperature. Membranes were washed and stored in PBS at 4°C until use. Ice cold methanol was added for 10 min at 4°C. PBS/1% (w/v) BSA/0.3% (w/v) Triton-X-100 (PBT) was used to block non-specific binding sites and permeabilize cells for 30 min at 4°C and specific binding sites were stained with mouse anti-HRV-A16 antibody (1:200; QED Bioscience, San Diego, CA, USA), anti-Mucin 5AC antibody (1:200; Thermo Fisher Scientific) or goat anti-FOXJ1 antibody (1:200; R&D, Minneapolis, MN, USA) for 1 h at room temperature. After washing, membrane was stained with donkey anti-rabbit, donkey anti-mouse, donkey anti-goat Alexa-flour antibodies (all were diluted in 1:200, Thermo Fisher Scientific) and 4′,6-diamidino-2-phenylindole (DAPI, 1:50, Sigma-Aldrich) in the dark for 30 min at room temperature. After that, membranes were placed on a glass slides covered with prolong gold anti fading reagent (Thermo Fisher Scientific) and with a coverslip. Slides were viewed using a Leica TCS SP8 confocal microscope (Leica Microsystems, Wetzlar, Germany) at 630 x original magnification.

### Statistical Analysis

Statistical analysis was performed in GraphPad PRISM 8.0 (GraphPad Software Inc., La Jolla, CA). Differences were explored by paired *t*-test, one- or two-way ANOVA with Tukey's test between groups. Data are shown as means ± SEM of cultures derived from several donors and differences were considered significant at *P* < 0.05.

## Results

### Effects of Epithelial Infection by HRV-A16 on Its Receptor and Epithelial Cell Markers

**Figure 1:**
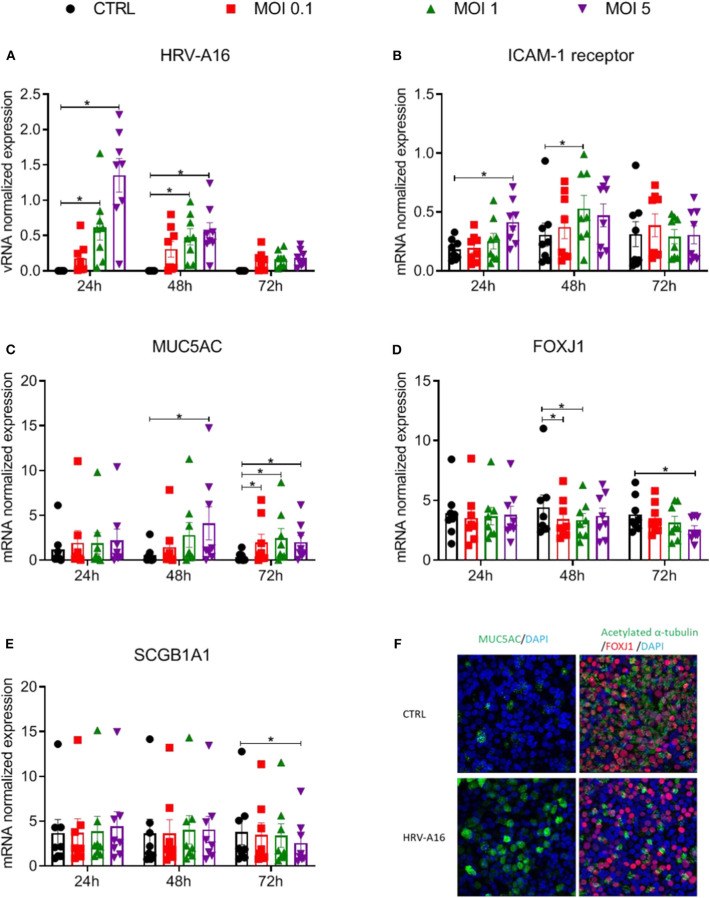
Effects of epithelial infection by HRV-A16 on its receptor and epithelial cell markers ALI-PBEC were infected with HRV-A16 (MOI 0.1, 1, 5) for 1 h and were incubated for 24, 48, or 72 h. **(A–E)** The replication of HRV-A16 vRNA and gene expression of ICAM-1, MUC5AC, SCGB1A1, and FOXJ1 were measured by qPCR. Data are shown as target gene expression normalized for RPL13A and ATP5B. Data are mean values ± SEM. *n* = 8 independent donors. Analysis of differences was conducted using two-way ANOVA with a Tukey *post-hoc* test. Significant differences are indicated by *P < 0.05 compared with control. **(F)** At 48 h after HRV-A16 infection, cells were fixed in 4% paraformaldehyde solution and stained using immunofluorescence with primary antibodies against MUC5AC (goblet cell markers, green) or acetylated α-tubulin and FOXJ1 (ciliated cell markers, green, and red) in combination with 4′,6-diamidino-2-phenylindole (DAPI) for nuclear staining (blue). Images shown are representative for results obtained with cells from four different donors with 630 x original magnification (*n* = 4).

ALI-PBEC were harvested at 24, 48, 72 h after viral infection. Viral RNA (vRNA) levels increased dose-dependently and peaked at 24 h (Figure 1A). HRV-A16 infection also caused an increase in ICAM-1, the receptor for the major group of HRV including HRV-A16 (Figure 1B). We next investigated whether HRV-A16 causes changes in epithelial cell markers. We used real time-qPCR to measure markers of ciliated cells (FOXJ1), goblet cells (MUC5AC) and club cells (SCGB1A1). HRV-A16 infection caused a significant and dose-dependent increase in MUC5AC gene expression (Figure 1C). This increase was accompanied by a dose-dependent decrease in FOXJ1 expression at both 48 and 72 h, and a decrease of SCGB1A1 at MOI 5 and 72 h after infection (Figures 1D,E). To confirm these findings at the protein level, an immunofluorescence analysis was performed. Consistent with our gene expression analysis, HRV-A16 infection resulted in an increase in marker of goblet cells (Mucin 5AC-positive cells) and a decrease in markers of ciliated cells (acetylated α-tubulin [structural protein] and FOXJ1 [transcription factor involved in ciliogenesis]) (Figure 1F).

**Figure 2:**
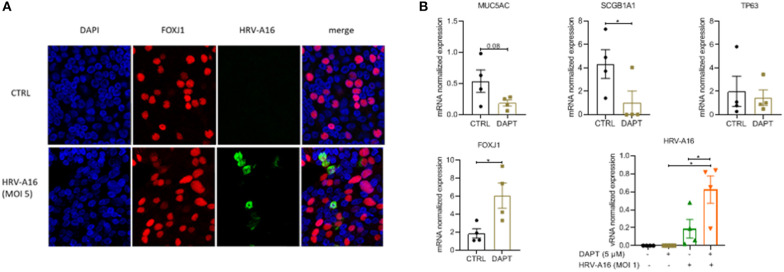
The target of HRV-A16 in human bronchial epithelial cells. **(A)** At 8 h after HRV-A16 infection, cells were fixed in 4% paraformaldehyde solution and stained using immunofluorescence with mouse anti-HRV-A16 antibody (green), primary antibodies against FOXJ1 (ciliated cell marker, red) in combination with 4′,6-diamidino-2-phenylindole (DAPI) for nuclear staining (blue). Images shown are representative for results obtained with cells from four different donors with 630 x original magnification (*n* = 4). **(B)** PBEC were differentiated for 14 days in the presence of either 5 uM DAPT or solvent control. On day 14, cells were infected by HRV-A16 (MOI 1). After infection, the basal medium was replaced by fresh medium contain 5 uM DAPT or solvent control. Cultures were incubated for 24 h before harvesting lysis of cells for isolation of RNA. The vRNA and gene expression of cell markers (MUC5AC, SCGB1A1, TP63, and FOXJ1) were measured by qPCR. Data are mean values ± SEM. Data are shown as target gene expression normalized for RPL13A and ATP5B. *n* = 4 independent donors. Analysis of differences was conducted using paired *t*-test and one-way ANOVA with a Turkey *post-hoc* test. Significant differences are indicated by *P < 0.01 compared with control or HRV-A16.

To investigate the impact of an altered composition of the airway epithelium on infection by rhinovirus, we used immunofluorescence to show colocalization of HRV-A16 with airway epithelial subtypes. The results showed that HRV-A16 mainly colocalized with FOXJ1-positive ciliated cells (Figure 2A). To further substantiate these findings in our model, we next used Notch signaling inhibition by DAPT to induce a shift toward ciliated cell differentiation. Cellular differentiation in presence of DAPT for 2 weeks caused a marked shift in epithelial cell differentiation as previously reported by us (Amatngalim et al., 2018), with a marked decrease in secretory cells (MUC5AC and SCGB1A1) and an increase ciliated cells (Figure 2B). We found that vRNA levels following infection were significantly higher in ALI-PBEC treated with DAPT (Figure 2B), supporting the notion that ciliated cells are a primary target for rhinovirus infection.

### HRV-A16 Modulates a Network of Genes Mediating Mucin Production in ALI-PBEC

**Figure 3:**
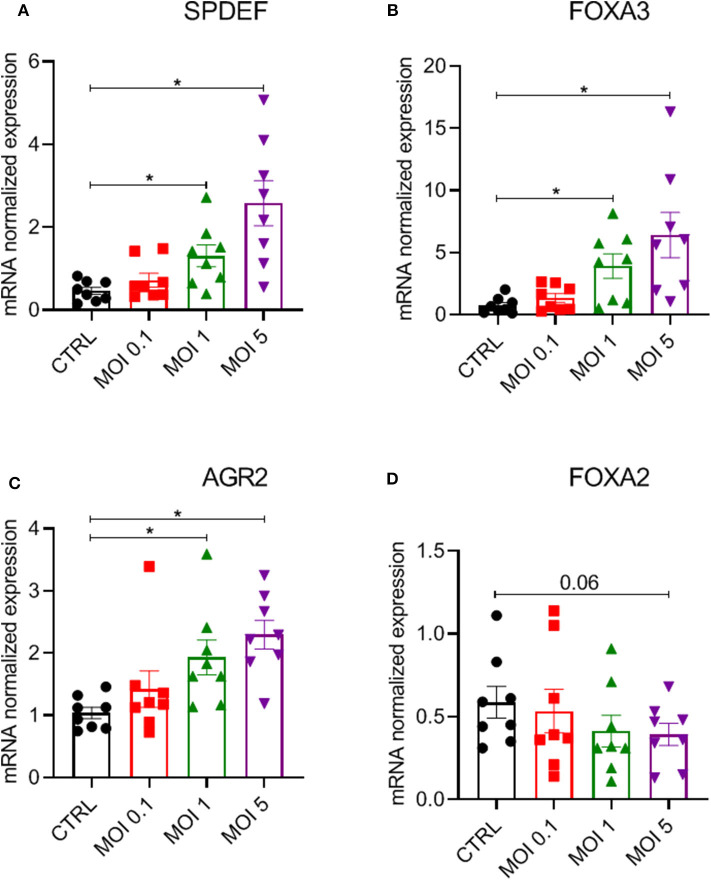
Modulation of a network of genes relating to SPDEF by HRV-A16. ALI-PBEC were infected with HRV-A16 (MOI 0.1, 1, 5). Cells were harvested at 48 h after infection. **(A–D)** The gene expressions of SPDEF, FOXA3, AGR, and FOXA2 were measured by real-time PCR Data are shown as target gene expression normalized for RPL13A and ATP5B. Data are mean values ± SEM. *n* = 8 independent donors. Analysis of differences was conducted by paired one-way ANOVA with a Tukey *post-hoc* test. Significant differences are indicated by *P < 0.05 compared with control.

To further investigate mechanisms underlying the increased epithelial mucin production following HRV-A16 infection, we explored pathways previously shown to be associated with epithelial mucin production. SPDEF has been implicated in the process of mucin production by regulating expression of a series of genes, including FOXA3, FOXA2, and AGR2. We next investigated role of HRV-A16 on SPDEF-regulated genes. Considering the time of high viral replication and mucin production, we chose 48 h as a key time for further experiments. At MOI 1 or 5 for HRV-A16, we found that gene expression of SPDEF, FOXA3, and AGR2 were significantly increased in ALI-PBEC at 48 h post-infection (Figures 3A–C). In contrast, HRV-A16 at MOI 5 caused a reduction in FOXA2 that did not reach significance (Figure 3D).

### Effects of Tiotropium and Fluticasone on Viral Infection in ALI-PBEC

To study effects of tiotropium and fluticasone on HRV-A16 replication and HRV-A16 induced mucin expression, first we identified effects of dosage. We used dose ranges of 10-1000 nM based on previous studies (Kistemaker et al., 2015; van den Berge et al., 2018). Neither tiotropium nor fluticasone affected vRNA levels, but fluticasone (and not tiotropium) did decrease HRV-A16-induced MUC5AC gene expression (Supplementary Figures 1A,B). Based on these observations and previous studies showing consistent effects at 10 nM on cultured airway epithelial cells (Kistemaker et al., 2015; Singanayagam et al., 2018), we selected 10 nM for further experiments.

**Figure 4:**
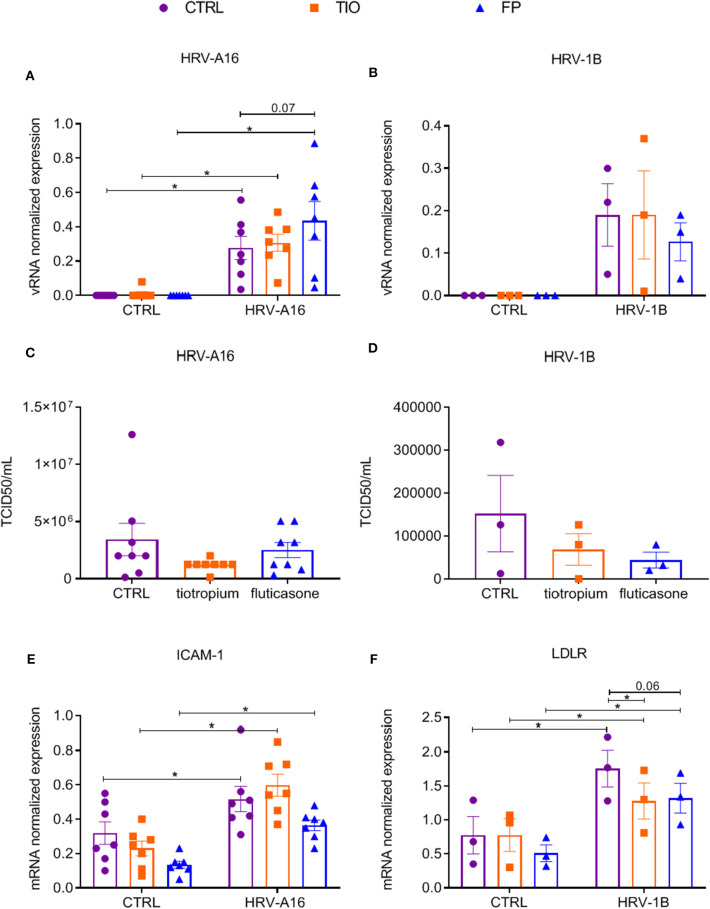
Effects of tiotropium bromide and fluticasone propionate on viral infection in ALI-PBEC. ALI-PBEC were pre-treated with tiotropium or fluticasone and infected with HRV-A16 (MOI 5). Cells were harvested at 48 h after infection. **(A,B)** The vRNA expression of HRV-A16 (*n* = 7) and HRV-1B (*n* = 3) together with **(C,D)** viral particles were measured by TCID50 assay (*n* = 8 or 3). **(E,F)** Gene expression of ICAM-1 (*n* = 7) and LDLR (*n* = 3) were examined by qPCR. Data are shown as target gene expression normalized for RPL13A and ATP5B or TCID50/mL. Data are mean values ± SEM. *n* = 3, 7, and 8 independent donors. Analysis of differences was conducted by two-way ANOVA with a Tukey *post-hoc* test Significant differences are indicated by *P < 0.05 compared with control or HRV-A16 group.

Having shown that fluticasone and tiotropium did not affect HRV-A16 vRNA levels and only fluticasone inhibited HRV-A16 produced MUC5AC expression, we evaluated effects of the minor group HRV-1B that uses low-density lipoprotein receptor (LDLR). At 48 h after infection, vRNA levels of neither HRV-A16 nor HRV-1B were affected by tiotropium and fluticasone although fluticasone showed a slight trend to enhance HRV-A16 vRNA expression (Figures 4A,B). In addition, drugs did not affect infectious viral particles in the apical wash as assessed by TCID50 assay (Figures 4C,D). Next, we showed that HRV-A16 and HRV-1B increased expression of their respective receptors ICAM-1 and LDLR (Figures 4E,F). No significant difference in expression of ICAM-1 were observed after treatment with tiotropium or fluticasone (Figure 4E). HRV-1B increased expression of LDLR was reversed with tiotropium or fluticasone (Figure 4F). Whereas, HRV-A16 infection caused an increase in LDH release, this was not modulated by tiotropium and fluticasone (Supplementary Figure 2).

### Effects of Tiotropium and Fluticasone on HRV-Induced Mucin Production in ALI-PBEC

**Figure 5:**
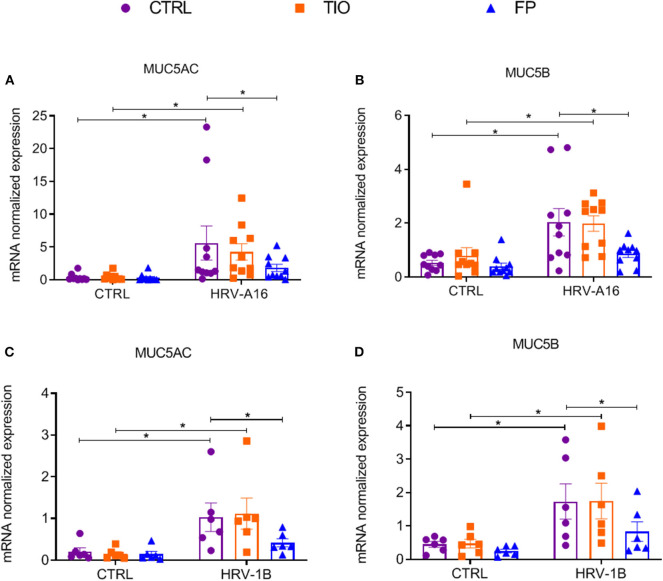
Effects of tiotropium and fluticasone on expression of MUC5AC and MUC5B in ALI-PBEC. ALI-PBEC were pre-treated with tiotropium or fluticasone and infected with HRV-A16 (MOI 5). Cells were harvested at 48 h after infection. The gene expression of MUC5AC **(A,C)** and MUC5B **(B,D)** were measured by real-time PCR. Data are mean values ± SEM. *n* = 10 or 6 independent donors. Data are shown as target gene expression normalized for RPL13A and ATP5B. Analysis of differences was conducted by two-way ANOVA with a Tukey *post-hoc* test Significant differences are indicated by *P < 0.05 compared with control or HRV-A16 group.

To further explore effects of tiotropium and fluticasone on HRV-induced mucin production, several experiments were performed. From gene expression analysis, we observed significantly enhanced expression of MUC5AC and MUC5B by both HRV-A16 and HRV-1B at MOI 5 in ALI-PBEC (Figure 5). Tiotropium and fluticasone did not contribute to changes in basal expression of these mucins in ALI-PBEC. However, the expression of both MUC5AC and MUC5B showed an evident decrease in ALI-PBEC infected by HRV-A16 or 1B and pre-treated by fluticasone but not tiotropium (Figure 5).

**Figure 6:**
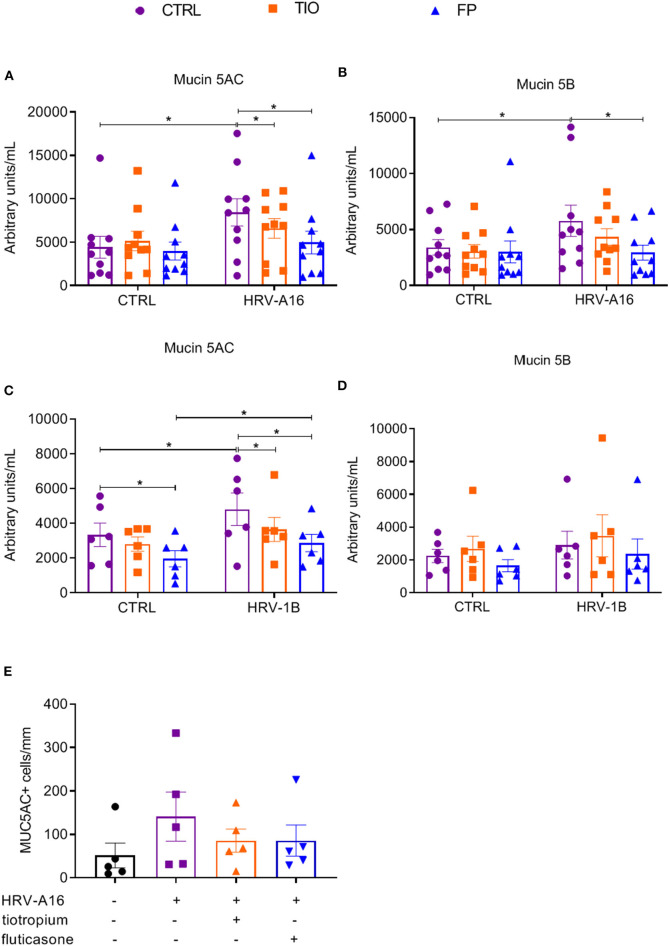
Effects of tiotropium and fluticasone on mucin release in ALI-PBEC. ALI-PBEC were pre-treated with tiotropium or fluticasone and infected with HRV-A16 (MOI 5). Cells were harvested at 48 h after infection. The protein levels of Mucin 5AC **(A,C)** and Mucin 5B **(B,D)** were measured by ELISA. *n* = 10 or 6 independent donors. **(E)** Mucin 5AC positive cells were quantified as positive cell numbers/mm (length of basal membrane, *n* = 4). Data are mean values ± SEM and shown as arbitrary units/mL according to standard line or MUC5AC+ cells/mm. Analysis of differences was conducted by paired one-way or two-way ANOVA with a Tukey *post-hoc* test Significant differences are indicated by *P < 0.05 compared with control or HRV-A16 group.

To better quantify mucin release, an ELISA assay was used to assess Mucin 5AC and Mucin 5B levels in apical wash of these cultures. Mucin 5AC levels were augmented by HRV-A16 or HRV-1B, which were counteracted by both tiotropium and fluticasone (Figures 6A,C). Elevated Mucin 5B levels were only elicited by HRV-A16, which was reversed by fluticasone (Figures 6B,D).

Furthermore, immunostaining and PAS staining were used to measure numbers of goblet cells and Mucin 5AC positive cells. It is plausible that goblet cell numbers were increasing in cultures treated with HRV but reduced by fluticasone and tiotropium (Supplementary Figure 3), which were further identified by immunostaining with Mucin 5AC antibody (Supplementary Figure 3). Although the number of Mucin 5AC+ cells appeared lower in infected cells treated with fluticasone or tiotropium, this difference was not significant (Figure 6E).

### Fluticasone Propionate Modulates HRV-Induced Genes Mediating Mucin Production in ALI-PBEC

**Figure 7:**
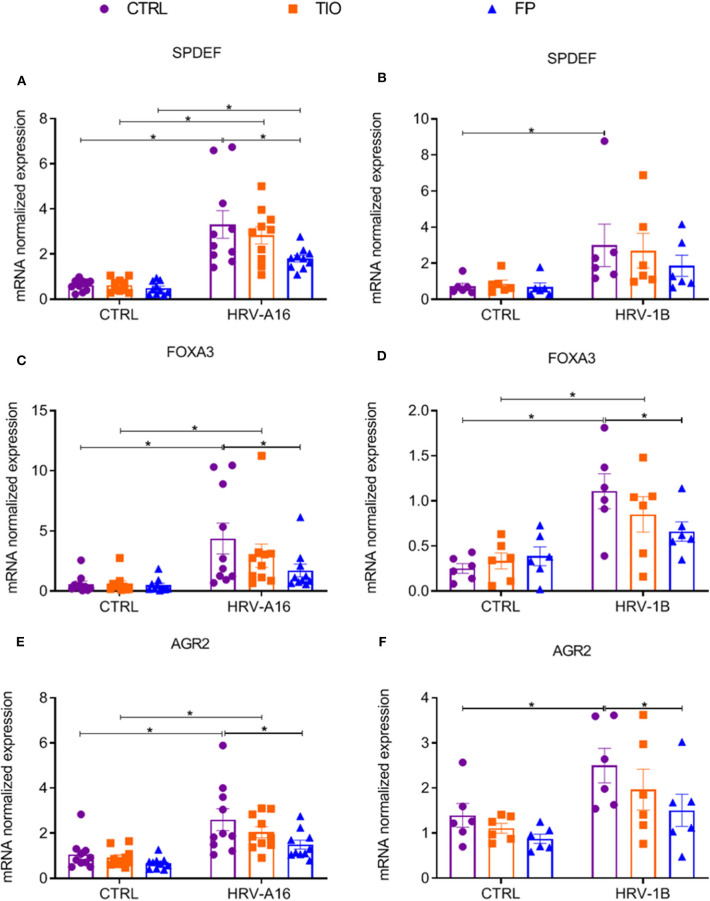
Modulation of fluticasone on SPDEF-regulated genes in ALI-PBEC. ALI-PBEC were pre-treated with tiotropium or fluticasone and infected with HRV-A16 (MOI 5). Cells were harvested at 48 h after infection. The gene expression of SPDEF **(A,B)**, FOXA3 **(C,D)** and AGR2 **(E,F)** were measured by real-time PCR. Data are shown as target gene expression normalized for RPL13A and ATP5B. Data are mean values ± SEM. *n* = 10 or 6 independent donors. Analysis of differences was conducted by two-way ANOVA with a Tukey *post-hoc* test Significant differences are indicated by *P < 0.05 compared with control or HRV-A16 group.

Since enhanced expression of SPDEF-regulated genes was found after HRV-treatment, we continued to investigate if tiotropium and fluticasone affected these genes. Fluticasone decreased HRV-augmented expression of the SPDEF and SPDEF-regulated genes (FOXA3 and AGR2) (Figure 7). Tiotropium and fluticasone did not show any effect on expression of HRV-induced FOXA2 expression even fluticasone caused a decrease in its basal expression (Supplementary Figure 4).

### Extracellular ATP Was Involved in Inhibition of Fluticasone on HRV-Induced Mucin Production

**Figure 8:**
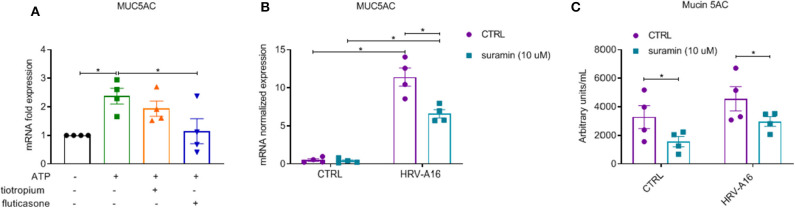
Inhibition of fluticasone on ATP-induced mucin production and involvement of extracellular ATP in HRV-induced mucin production and release in ALI-PBEC. **(A)** ATP (100 μM) treated cells in the presence of tiotropium or fluticasone. Cells were incubated for 24 h. The gene expression of MUC5AC was measured using qPCR. **(B)** ALI-PBEC were infected by HRV-A16 (MOI 5) after pre-treatment with suramin (10 μM). Cells were incubated for 48 h after infection. The gene expression of MUC5AC was measured by qPCR. **(C)** Levels of Mucin 5AC were assessed by ELISA assay. Gene expression were normalized for RPL13A and ATP5B. Data are shown as normalized or fold expression. Data are mean values ± SEM. *n* = 4 independent donors. Analysis of differences was conducted with paired one-way or two-way ANOVA with a Tukey *post-hoc* test Significant differences are indicated by *P < 0.05 compared with control, HRV-A16 and ATP group.

After 24 h stimulation with extracellular ATP, increased expression of MUC5AC was observed, which was inhibited by fluticasone not tiotropium (Figure 8A). To study role of purinergic signaling via extracellular ATP in HRV-induced mucin production and secretion, we chose suramin, a non-selective P2R antagonist to block this signaling based on previous research (Shishikura et al., 2016). We observed that HRV-induced MUC5AC gene expression and Mucin 5AC release was significantly inhibited by suramin as assessed by RT-PCR and ELISA analysis (Figures 8B,C).

## Discussion

The present study demonstrates that rhinovirus increases mucin production in differentiated cultures of primary human bronchial epithelial cells (ALI-PBEC), and that this increase may involve signaling via multiple pathways, including SPDEF-regulated genes and purinergic signaling via extracellular ATP in ALI-PBEC. Fluticasone inhibited HRV-augmented mucin production, which was accompanied by modulation of SPDEF and SPDEF-regulated genes and a decrease in ATP-induced MUC5AC gene expression. Most inhibitory effects of tiotropium did not reach statistical significance. These data are in line with the notion that HRV infection contributes to mucus hypersecretion during acute exacerbations in asthma and COPD, and demonstrate the potential of inhaled corticosteroids and anticholinergics to modulate these effects.

In the present study, we first assessed kinetics of HRV infection at different MOI. These results showed a rapid increase in HRV-A16 vRNA which peaked at 24 h, followed by a rapid clearance. Infection was accompanied by an increased dose-dependent expression of its receptor, ICAM-1. Next, we showed that HRV-A16 infection increased mucin gene expression and protein release. We assessed effects of HRV-A16 on epithelial composition, since epithelial remodeling has been suggested to explain increased susceptibility of e.g., infants to various stimuli (Jamieson et al., 2015). In line with previous findings (Hewson et al., 2010), expression of MUC5AC peaked at 48 h and was accompanied by a decrease in the ciliated cell marker FOXJ1. Expression of the club cell-marker SCGB1A1 was also decreased by HRV-A16, but only at 72 h post-infection. To further support these findings, we used confocal microscopy to demonstrate that HRV-A16 infection caused an increase in goblet cells and a decrease in ciliated cells. Together with other findings (Hayashi et al., 2004; Turner et al., 2011), this raises the possibility that ciliated and club cells may have acted as progenitors by transdifferentiation into goblet cells. Since we did not perform double-staining for goblet and ciliated cell markers, we do not know whether HRV-A16 may have resulted in the formation of mucociliary epithelial cells, as recently described in asthma (Vieira Braga et al., 2019).

Since we found HRV infection to cause changes in the cellular composition of the airway epithelium, and the epithelium is the primary site of HRV infection, we examined which cell type was the main target for HRV-A16. Previous studies have reported various epithelial cell types to be the primary site of infection with HRVs (Lachowicz-Scroggins et al., 2010; Jakiela et al., 2014; Griggs et al., 2017), Jakiela et al. and Griggs et al. identified ciliated cells as the main target for both HRV-A16 and HRV-C15, whereas Lachowicz-Scroggins et al. showed that HRV-A16 also infected goblet cells. To this end, we first used immunofluorescence co-staining to study the co-localization of HRV-A16 and next used the Notch signaling inhibitor DAPT to cause a shift toward an increase in ciliated cells. Collectively, these studies confirmed that ciliated cells are more sensitive to HRV infection. Notch signaling has also been implicated in HRV-induced production, but in contrast to previous findings (Jing et al., 2019), we could not confirm that HRV-A16 affected expression of the Notch receptors NOTCH1 and NOTCH3 (Supplementary Figure 5).

We next found that HRV-A16-induced mucin production was accompanied by an increase in expression of SPDEF, FOXA3, and AGR2, suggesting involvement of SPDEF and SPDEF-regulated genes. However, how HRV-A16 increases expression of these genes and how they are involved in HRV-induced mucin production is not clear, and further studies are needed. We also studied expression of genes associated with mucus hypersecretion that have not been previously investigated in culture models of HRV infection. Calcium-activated chloride channel (transmembrane protein 16A, TMEM16A) and calcium-activated chloride channel regulator 1 (CLCA1, the human ortholog of mouse CLCA3/Gob-5) have both been linked to mucus hypersecretion in asthma and COPD (Brett, 2015). CLCA1 is a secreted protein activating currents through endogenous Ca^+^-activated Cl^−^ channels (CaCCs, such as TMEM16A) (Sala-Rabanal et al., 2015). We found that HRV-A16 increased expression of CLCA1 but not TMEM16A (Simoes et al., 2019) (Supplementary Figure 6). Since previous studies showed that IL-13 does increase expression of CaCCs (Lin et al., 2015; Qin et al., 2016), these findings indicate that pathways leading to increased mucin expression induced by HRV and IL-13 differ. This is further supported by the fact that we found that HRV-A16 also increased MUC5B, whereas in the same culture system we found that IL-13 decreased MUC5B (Mertens et al., 2017). Other studies reported on the involvement of other mechanisms in virus-induced mucin hypersecretion. Double-stranded RNA poly(I:C) and type 14 rhinovirus (HRV-14) have been shown to regulate the increased secretion and production of mucins through release of extracellular ATP signaling via purinergic P2 receptors (P2Rs) (Shishikura et al., 2016). In the present study, we confirmed that ATP induced MUC5AC expression and found that blocking purinergic signaling reversed HRV-induced MUC5AC expression and Mucin 5AC release. Thereby, our study using primary differentiated airway epithelial cells confirms the findings of Shishikura et al. obtained in the pulmonary mucoepidermoid carcinoma cell line NCI-H292. Collectively, we provide evidence to suggest that HRV increases mucin production and secretion partly through SPDEF-regulated genes and extracellular ATP-mediated purinergic signaling.

After delineating mechanisms underlying HRV-increased mucin expression, we next investigated potential modulation of this process by drugs used in the treatment of asthma and COPD, and focused on the LAMA tiotropium and the ICS fluticasone. Tiotropium and fluticasone did not modulate infection, nor HRV-induced expression of the major group HRV receptor ICAM-1, whereas tiotropium and fluticasone did cause a limited decrease in HRV-induced expression of LDLR, the receptor for minor group HRV. These findings are in contrast to those by Yamaya et al., who previously reported that tiotropium reduces HRV-14 titres, vRNA and ICAM-1 receptor (Yamaya et al., 2012). This may in part be explained by the observation that HRV-14 infection in their study was not cleared from the tracheal epithelial cell cultures, whereas in our study HRV-A16 replication was highest at 24 h and cleared quickly in ALI-PBEC. In line with our findings, another study also did not find an effect of fluticasone on viral levels at 48 h, but did find that fluticasone impaired viral clearance at 72 h post-infection (van den Berge et al., 2018). Furthermore, one study showed that fluticasone might even increase viral replication at later time points after infection (Thomas et al., 2014). Next, we evaluated effects of tiotropium and fluticasone on HRV-induced mucin production. We found that tiotropium had a partial inhibitory effect on HRV-A16 increased mucin 5AC protein levels, but not on MUC5AC gene expression, which was in line with our previous observations showing that tiotropium significantly decreased IL-13-increased mucin producing cells without a significant effect on MUC5AC gene expression (Kistemaker et al., 2015). This finding is however in contrast to that from another study, showing a significant inhibitory effect of tiotropium on neutrophil elastase-induced MUC5AC gene expression (Komiya et al., 2017). Our data suggest that the effect of tiotropium on mucin release and goblet cell numbers may be more pronounced than its effect on mucin gene expression. However, limited donor numbers, donor variation and other study-related parameters may also explain why we did not see effects of tiotropium on mucin gene expression. Tiotropium did not affect HRV-A16 induced changes in SPDEF-regulated genes and in extracellular ATP-mediated purinergic signaling, indicating that tiotropium decreased mucin 5AC protein levels through other pathways. Although we could not pinpoint the mechanism by which tiotropium decreased HRV-induced mucin 5AC levels, we did find that HRV enhanced muscarinic M3 receptor expression (Supplementary Figure 7), which is inhibited by the M3 specific muscarinic receptor antagonist tiotropium and activated by acetylcholine, supporting a role for acetylcholine in the regulation of mucin production (Gosens and Gross, 2018). In contrast to these findings with tiotropium, in our study fluticasone inhibited both HRV-induced mucin gene expression and protein production, which appears inconsistent with recent findings in other studies. Based on studies in a mouse model and in cell culture, Singanayagam et al. concluded that inhaled corticosteroids increase mucus production during COPD exacerbations (Singanayagam et al., 2018). Differences with our findings may be explained by the use of a mouse model and of cells from COPD patients at stage III, whereas the majority of donors used in the present study had a normal lung function. This latter may be especially important, since it has been reported that HRV-mediated mucin expression in airway epithelial cells, derived from COPD patients, may involve a different pathway (Jing et al., 2019). Furthermore, also the HRV species used were different: they used minor group HRV-A1, whereas major group HRV-A16 and minor group HRV-1B were used in our studies. Importantly, in contrast to the absence of an effect of fluticasone on viral replication in our study, they found that fluticasone increased HRV-A1 replication, which can also partly explain the observed increased mucin production in fluticasone-treated infected cells. We further investigated possible mechanisms underlying modulation of fluticasone on HRV-induced mucin production. Fluticasone inhibited HRV-induced expression of SPDEF and SPDEF-regulated genes. This inhibition by fluticasone was also found in IL-13 treated cells and in patients with Th2-high asthmatics (Lachowicz-Scroggins et al., 2017). Fluticasone also reduced ATP-induced MUC5AC expression, which indicated that fluticasone modulates ATP-mediated purinergic signaling. Further experiments need to be done to confirm this finding in the future. Recent evidence suggests mucin-specific therapeutic approaches could be of benefit because complete MUC5B removal from the airway may be detrimental, whilst the identification of MUC5AC as an essential non-contractile mediator of airway hyperresponsiveness and the role of MUC5AC in tethering mucus plugging suggest that MUC5AC-specific therapies could be of benefit in asthma (Livraghi-Butrico et al., 2017). In this respect, it is important to note that we found that fluticasone inhibited both MUC5AC and MUC5B, whereas tiotropium only affected Mucin 5AC release. Whether the combination of tiotropium and fluticasone has different effects on HRV replication and HRV-induced mucin expression than separate treatment requires further studies.

Collectively, we show that HRV infection increases epithelial mucin production and secretion in well-differentiated bronchial epithelial cells, and provide evidence that modulation of SPDEF-regulated genes and ATP-mediated purinergic signaling may contribute to these effects. Our findings suggest that fluticasone decreased HRV-induced mucin production partly by counteracting the HRV-induced expression of SPDEF-regulated genes and ATP-mediated purinergic signaling. In contrast, tiotropium caused a decrease in HRV-increased Mucin 5AC release, but did not affect gene expression. These findings provide further insight into the mechanisms underlying the clinical benefit of using tiotropium and fluticasone in the treatment of asthma and COPD, and additional insight into the mechanisms by which HRV increases epithelial mucin production.

## Data Availability Statement

The datasets generated for this study are available on request to the corresponding author.

## Ethics Statement

Ethical review and approval was not required for the study on human participants in accordance with the local legislation and institutional requirements. Written informed consent for participation was not required for this study in accordance with the national legislation and the institutional requirements.

## Author Contributions

Conception and design and analysis, interpretation, and drafting the manuscript for important intellectual content: YW and PH. Performance of experiments: YW, DN, and AS. All authors have read the manuscript and approve its submission.

## Conflict of Interest

The authors declare that the study was supported by a research grant from Boehringer Ingelheim, the manufacturer of tiotropium used in the present study. No other conflicts of interest are reported.
