# Supplementary material for: Tiotropium and Fluticasone Inhibit Rhinovirus-Induced Mucin Production via Multiple Mechanisms in Differentiated Airway Epithelial Cells
Source: Front Cell Infect Microbiol. 2020 Jun 19;10:278. doi: 10.3389/fcimb.2020.00278 (PMC7318795; doi:10.3389/fcimb.2020.00278)
Supplement: Supplementary file 1 [file Data_Sheet_1.docx]

Supplementary Material

# Supplementary Methods

## LDH assay

Apical wash and basal medium were centrifuged directly after harvesting. Diluted samples and positive control (100% lysate, apical wash from 0.1% (w/v) Triton X-100/PBS-treated cells) were mixed with reaction mixture for 30 min in a 96-well plate according to the manufacturer’s instructions (Cytotoxicity detection kit; Roche, Germany). OD values were measure at 490 nm and results were calculated as % Cytotoxicity = 100 x Sample LDH release/Maximum LDH.

# Supplementary Figures and Tables

## Supplementary Figures


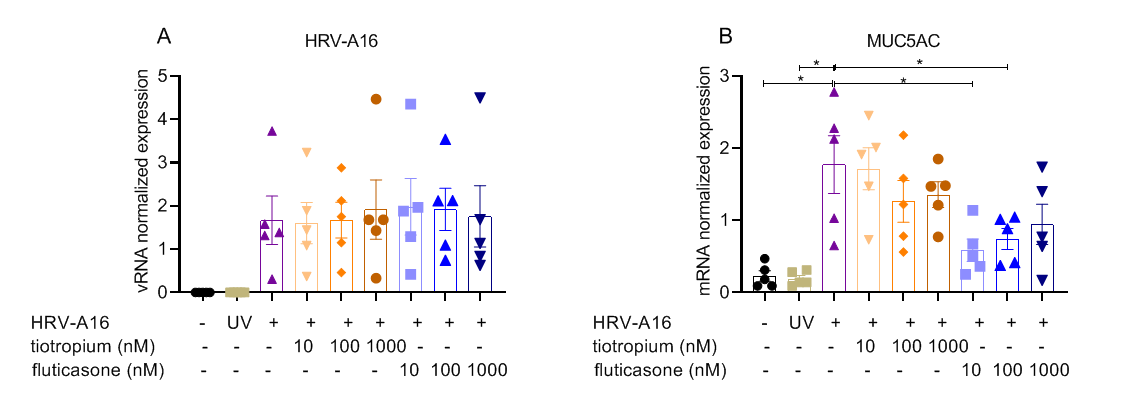


Supplementary Figure 1. Effects of tiotropium and fluticasone on viral replication and HRV-augmented MUC5AC expression. ALI-PBEC were pre-treated with either tiotropium/fluticasone or control, and exposed to HRV-A16 (MOI 5) or UV-treated HRV-A16 for 1 h. Next, apical HRV-A16 was removed, and basal medium was changed in complete medium (no HC) in the presence of tiotropium/fluticasone. Cells were harvested at 48 h after infection. (A, B) The vRNA levels and gene expression of MUC5AC were measured by qPCR. Data are shown as target gene expression normalized for RPL13A and ATP5B. Data are mean values ± SEM. n=5 independent donors. Analysis of differences was conducted by two-way ANOVA with a Tukey post-hoc test. Significant differences are indicated by *P<0.05 compared with control or HRV-A16 group.


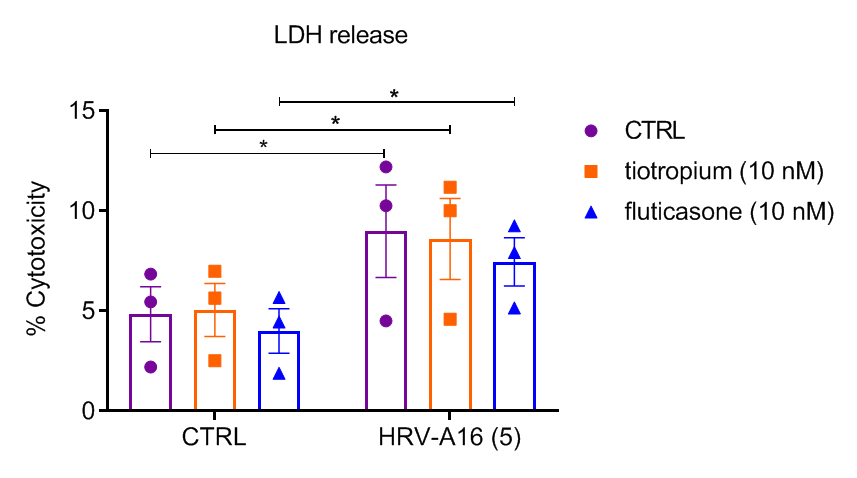


**Supplementary Figure 2. Cytotoxicity measurement with LDH assay.** ALI-PBEC were pre-treated with tiotropium or fluticasone and infected with HRV-A16 (MOI 5). Apical wash and basal medium were harvested at 48 h after infection. Percentage cytotoxicity was calculated as 100 x Sample LDH release/Maximum LDH. Data are mean values ± SEM. n=3 independent donors. Significant differences are indicated by *P<0.05 compared with control or HRV group.


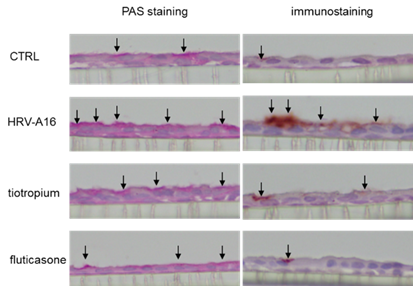


**Supplementary Figure 3. PAS staining and immunostaining assays for Mucin 5AC+ cells.** Transwells with cells were fixed by 4% (w/v) paraformaldehyde in PBS. Membranes were embedded in 2% (w/v) agar (VWR) prior to paraffin embedding and cut into 4 µm thick slices for periodic acid-Schiff (PAS) staining (left) and immunostaining (right). n=4 independent donors.


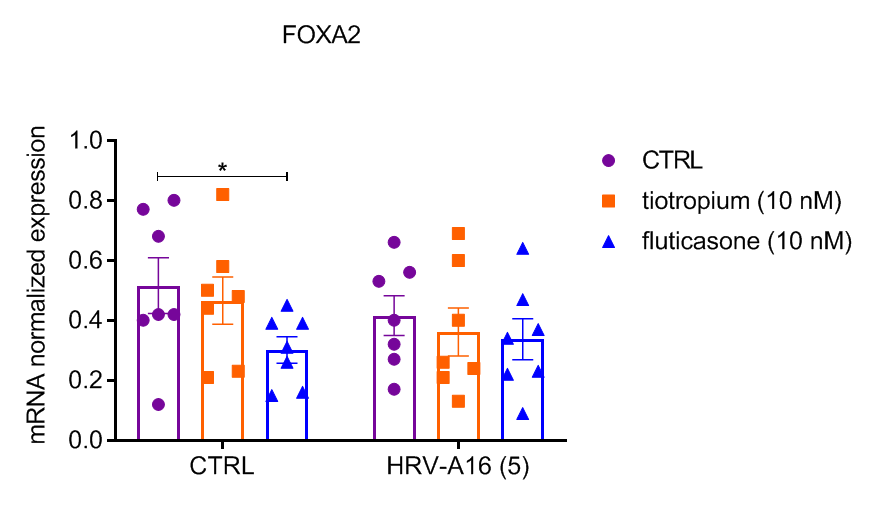


**Supplementary Figure 4. Effect of tiotropium and fluticasone on HRV-induced FOXA2 expression in ALI-PBEC.** ALI-PBEC were pre-treated with tiotropium or fluticasone and infected with HRV-A16 (MOI 5). Cells were incubated for 48 h. Gene expression of FOXA2 was examined by qPCR. Data are shown as target gene expression normalized for RPL13A and ATP5B. Data are mean values ± SEM. n**=**7 independent donors. Significant differences are indicated by *P<0.05 compared with control or HRV group.

**Supplementary Figure 5. Expression of Notch receptors NOTCH 1 and 3 in HRV-A16 infected human bronchial epithelial cells.** ALI-PBEC were infected with HRV-A16 (MOI 0.1, 1, 5). Cells were harvested at 48 h after infection. The gene expression of NOTCH 1 and NOTCH 3 were measured by real-time qPCR (n=8). Data are shown as target gene expression normalized for RPL13A and ATP5B. Data are mean values ± SEM. n=4, 8 independent donors. Significant differences are indicated by *P<0.05 compared with control or HRV group.


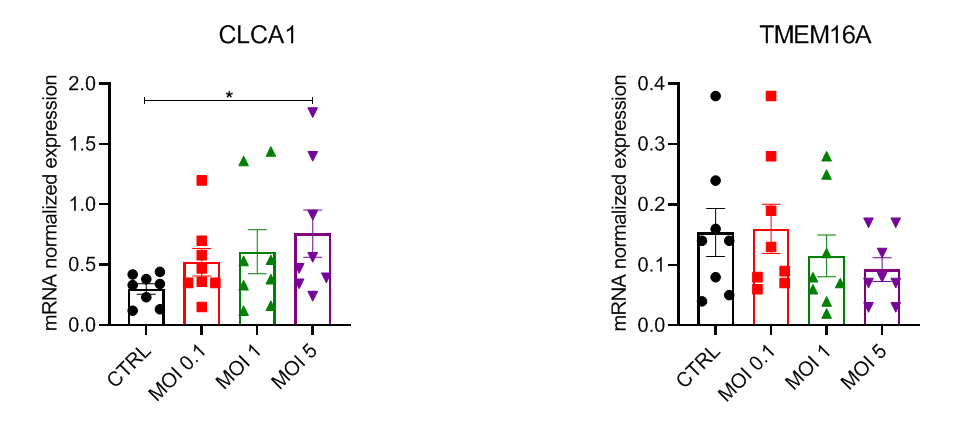


B

A

Supplementary Figure 6. CaCC-associated gene expression in HRV-A16 infected ALI-PBEC. ALI-PBEC were infected with HRV-A16 (MOI 0.1, 1, 5). Cells were harvested at 48 h after infection. Gene expression of CLCA1 (A) and TMEM16A (B) was measured by real-time PCR. Data are shown as target gene expression normalized for Ribosomal Protein L13a (RPL13A) and ATP synthase, H+ transporting, mitochondrial F1 complex, beta polypeptide (ATP5B). Data are mean values ± SEM. n=8 independent donors. Significant differences are indicated by *P<0.05 compared with control.


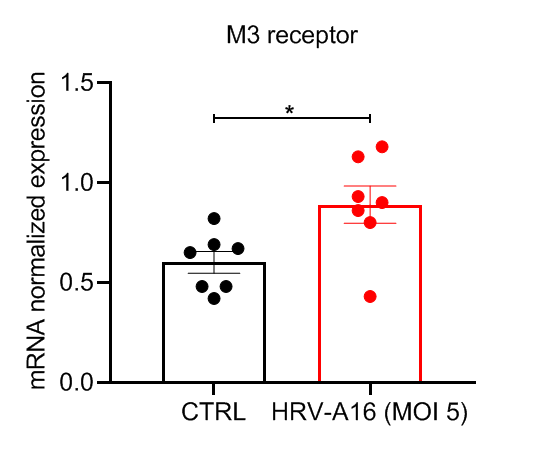


Supplementary Figure 7. Expression of muscarinic 3 receptor in HRV-A16 infected ALI-PBEC. ALI-PBEC were infected with HRV-A16 (MOI 5) and harvested at 48 h after infection. The gene expression of M3 receptor was measured by real-time PCR. Data are shown as target gene expression normalized for Ribosomal Protein L13a (RPL13A) and ATP synthase, H+ transporting, mitochondrial F1 complex, beta polypeptide (ATP5B). Data are mean values ± SEM. n=7 independent donors. Significant differences are indicated by *P<0.05 compared with control.
